# P-1388. Repeat *Trichomonas vaginalis* Infections Among Pregnant Women in South Africa

**DOI:** 10.1093/ofid/ofae631.1564

**Published:** 2025-01-29

**Authors:** Lindsay S Lim, Mandisa Mdingi, Freedom Mukomana, Ranjana M S Gigi, Nicola Low, Remco Peters, Andrew Medina-Marino, Jeffrey Klausner, Christina A Muzny

**Affiliations:** University of Alabama at Birmingham, AdventHealth, Orlando, Florida; Foundation for Professional Development, Research Unit, East London, Eastern Cape, South Africa; Foundation for Professional Development, East London, Eastern Cape, South Africa; Foundation for Professional Development, Research Unit; University of Bern, Department of Social and Preventative Medicine, East London, Eastern Cape, South Africa; University of Bern, Bern, Bern, Switzerland; University of Pretoria, Department of Medical Microbiology; 5. University of Cape Town, Division of Medical Microbiology, East London, Eastern Cape, South Africa; Desmond Tutu HIV Centre, University of Cape Town; Perelman School of Medicine, University of Pennsylvania, East London, Eastern Cape, South Africa; Keck School of Medicine of USC, Los Angeles, California; University of Alabama at Birmingham, Birmingham, AL

## Abstract

**Background:**

*Trichomonas vaginalis* (TV) is the most common non-viral sexually transmitted infection (STI) worldwide. It has been associated with multiple adverse outcomes in pregnancy. The prevalence of repeat TV infections among pregnant women is poorly characterized, especially in resource-limited countries. This study aimed to investigate the prevalence and causes of repeat TV infections in pregnant women in South Africa.

**Figure:**
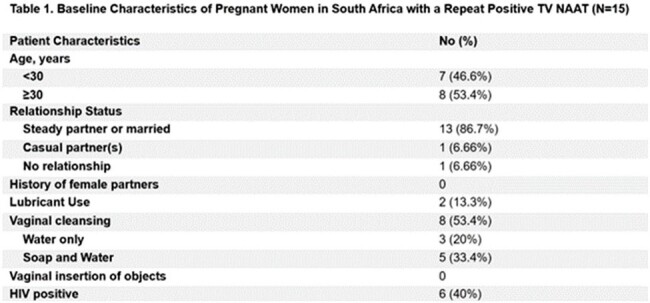

**Methods:**

Women enrolled in a randomized controlled trial investigating optimal STI screening strategies during pregnancy were included. At baseline, TV was diagnosed via the GeneXpert nucleic acid amplification test (NAAT). Demographics, sexual history, and partner characteristics were collected. If positive, women were treated with multi-dose oral metronidazole and randomized to Arm 1 or 2 (test-of-cure at 3 weeks after enrollment or re-testing at 32-34 weeks gestation). At follow-up in both arms, a repeat TV NAAT was obtained and a questionnaire was administered examining adherence to treatment, partner treatment history, and interval sexual activity. Women with confirmed treatment completion and a repeat positive TV NAAT were classified as treatment failure if no interval sexual activity, re-infection if had interval sexual exposure to baseline partners who were not treated as contacts, or new infection if exposed to a new partner.

**Figure:**
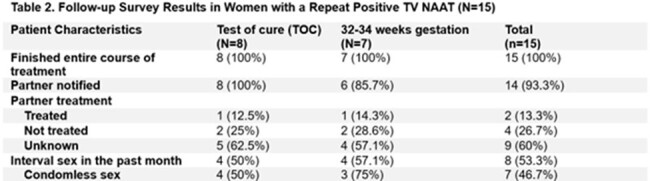

**Results:**

Baseline TV prevalence was 10% (144/1440) (Figure 1) and 10.4% (15/144) of infected women had a repeat positive test with baseline characteristics shown in Table 1. All 15 women finished their entire course of treatment and partners were notified in 93.3% of cases (Table 2). Based on our definitions, 60% of women (9/15) were classified as treatment failure in the setting of no interval sexual exposure or sexual exposure with partner who was reportedly treated (Figure 2). 26.7% (4/15) were classified as re-infection and 13.3% (2/15) were unable to be classified. There were no new infections.

**Figure:**
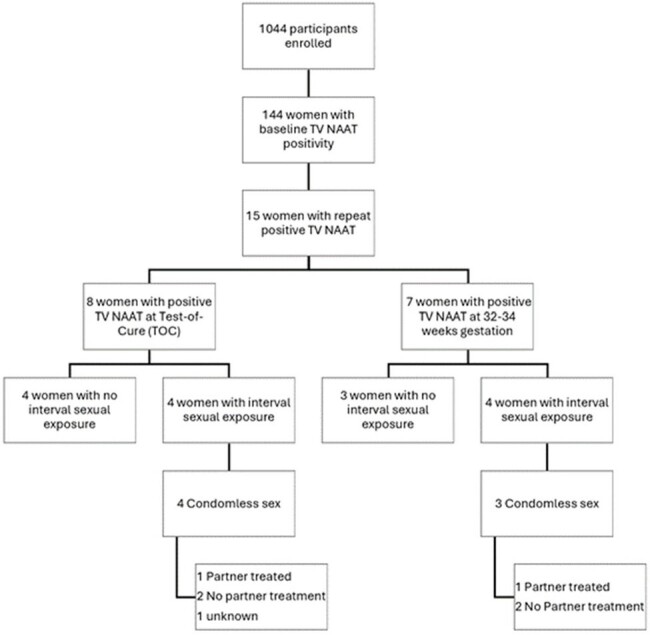

**Conclusion:**

More than half of repeat TV infections in this cohort of pregnant women in South Africa were determined to be due to treatment failure. Additional studies with larger sample sizes are needed to further investigate potential causes and include drug susceptibility testing on clinical TV isolates in women with repeat infections.

**Figure:**
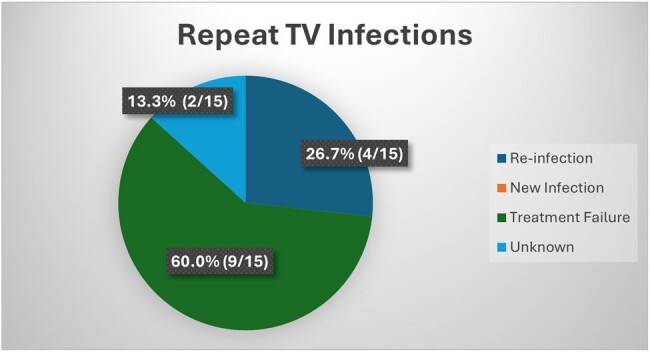

**Disclosures:**

**Jeffrey Klausner, MD MPH**, Direct Diagnostics: Advisor/Consultant **Christina A. Muzny, MD, MSPH**, Abbott: Advisor/Consultant|Abbott: Grant/Research Support|BioMed Diagnostics: Advisor/Consultant|BioNTech: Advisor/Consultant|BioNTech: Grant/Research Support|Cepheid: Advisor/Consultant|Cepheid: Honoraria|Elsevier: Honoraria|Gilead: Grant/Research Support|Lupin Pharmaceuticals: Grant/Research Support|Merck Manuals: Board Member|Merck Manuals: Honoraria|UpToDate: Honoraria|Visby: Grant/Research Support

